# The Evaluation of Sandwich Composite Materials with Vegetable Fibers in a Castor Oil Polyurethane Matrix with Their Faces and Honeycomb Core Made in a 3D Printer

**DOI:** 10.3390/polym16212980

**Published:** 2024-10-24

**Authors:** Gilberto Garcia del Pino, Abderrezak Bezazi, Antonio Claudio Kieling, José Costa de Macedo Neto, Sofia Dehaini Garcia, José Luis Valin Rivera, Meylí Valin Fernández, Aristides Rivera Torres, Francisco Rolando Valenzuela Diaz

**Affiliations:** 1 Department of Mechanical Engineering, Higher School of Technology, Amazonas State University, Manaus 69050-020, Brazil; akieling@uea.edu.br (A.C.K.); jmacedo@uea.edu.br (J.C.d.M.N.); artorres@uea.edu.br (A.R.T.); 2 Laboratoire de Mécanique Appliquée des Nouveaux Matériaux (LMANM), University of Guelma, Guelma 24000, Algeria; ar_bezazi@yahoo.com; 3 Department of Mechanical Engineering, Federal University of São João del Rei, São João del-Rei 36307-352, Brazil; sofidehaini@gmail.com; 4 Escuela de Ingeniería Mecánica, Pontificia Universidad Católica de Valparaíso, Valparaíso 2340025, Chile; jose.valin@pucv.cl; 5 Department of Mechanical Engineering (DIM), Faculty of Engineering (FI), University of Concepción, Concepción 4030000, Chile; mvalin@udec.cl; 6 Department of Materials Engineering and Metallurgy, University of São Paulo, São Paulo 05508-220, Brazil; frrvdiaz@usp.br

**Keywords:** sisal, PLA, PETG, 3D printing, sandwich panel, composite

## Abstract

Sandwich panels are widely used in the naval and aerospace industries to withstand the normal tensile, compressive, and shear stresses associated with bending. The faces of sandwich composites are usually made of metals such as aluminum and, in some studies with composites, using a polymeric matrix, but there are no studies in the literature using a castor oil polyurethane matrix. The core of the panel must keep the faces apart and be rigid perpendicular to them. To begin the work, a study was carried out on the influence of alkaline treatment on sisal fibers to increase the fibers’ adhesion to castor oil polyurethane. There are no relevant studies worldwide on the use of this resin and the adhesion of vegetable fibers to this polyurethane. In this work, a study was carried out through a three-point bending test of sandwich panels using faces of composite material with sisal fibers subjected to an alkaline treatment of 10% by weight of sodium hydroxide and an immersion time of 4 h in the dissolution, which was the best chemical treatment obtained initially in a castor oil polyurethane matrix. The honeycomb cores were made by 3D printer and in this study two different printing filament materials, PETG and PLA, and two different core heights were compared. As a result of a traction test, it was observed that sisal fibers with chemical treatment in a castor oil polyurethane matrix can be used in composites, although the stress levels obtained are 50% lower than the stresses obtained in other matrixes such as epoxy resin. The combination of sisal faces in a castor oil polyurethane matrix and honeycomb cores made in a 3D printer showed good properties, which allows the use of renewable, sustainable and less aggressive materials for the environment. In all tests, PETG was 21% to 32% stronger than PLA. Although there was no rupture in the test specimens, the PETG cores deformed 0.5% to 3.6% less than PLA. The composites with PLA were lighter, because the core density was 13.8% lower than the PETG cores. Increasing the height of the honeycomb increased its strength.

## 1. Introduction

Plant fibers are increasingly being explored as promising reinforcements in the composition of composite materials. This growing interest is attributed to their low environmental impact, affordability, wide availability, versatile applications (such as in construction, packaging, furniture, and transportation), low energy consumption, and minimal health risks [1,2,3]. Additionally, the biodegradability of these fibers offers a solution to mitigate environmental pollution [4,5].

Natural fibers are known for their versatility and abundance as they can be sourced from a wide variety of plants, trees, crops, and agricultural waste. Incorporating natural fibers into cementitious composites also slows the release of carbon into the atmosphere [6,7]. These fibers have the potential to enhance the toughness, ductility, flexural capacity, and crack resistance of cementitious composites [8,9]. Among the many types of natural fibers, sisal is increasingly cultivated worldwide, which reduces transportation needs and, consequently, carbon dioxide emissions. Sisal fibers are highly valued for their strength and biodegradability, making them a viable source of natural reinforcement [10]. Additionally, sisal fibers offer better abrasion and fungal resistance, improved thermal stability, and are considered economically advantageous and environmentally friendly [11,12].

**Figure 1:**
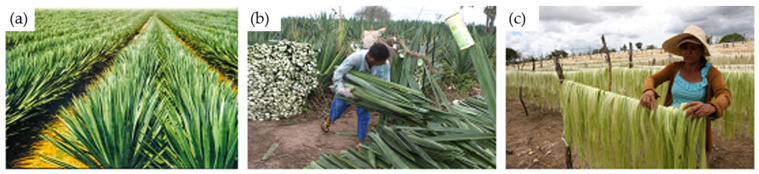
Sisal fiber processing in the State of Bahia, Brazil: (**a**) sisal planting, (**b**) collection, and (**c**) the drying of the fiber extracted from the leaves [13].

The sisal plant belongs to the Agave sisalana family, and its fibers are extracted from the leaves, which can grow up to 2 m in length, as you can see in Figure 1. The number of fiber bundles per leaf depends on the age and origin of the plant [4,13]. The properties and chemical compositions can be seen in Table 1.

The main issue with polymer composites reinforced with plant fibers is the incompatibility between the fibers and the polymer matrix. This incompatibility arises because the fibers are hydrophilic, as they are derived from lignocellulose, which contains strongly polarized hydroxyl groups, while the polymer matrix is hydrophobic. As a result, there is weak interfacial adhesion between the polar, hydrophilic fibers and the non-polar, hydrophobic matrix. Additionally, difficulties in mixing occur due to the low wettability of the fibers by the matrix, leading to a weak interface in these composites and, consequently, a reduction in the mechanical strength of the composite [15].

Therefore, the fibers must be properly modified through physical or chemical treatments to enhance their adhesion to the matrix [14,15], thereby improving the mechanical strength of the composite. One of the most commonly used is the alkaline treatment with sodium hydroxide (NaOH). The effect of alkaline treatment with NaOH—in natural fibers—on the dynamic and static performance of the composite has been shown to yield excellent results, particularly in terms of maximum flexural and tensile strength [12,14].

**Figure 2:**
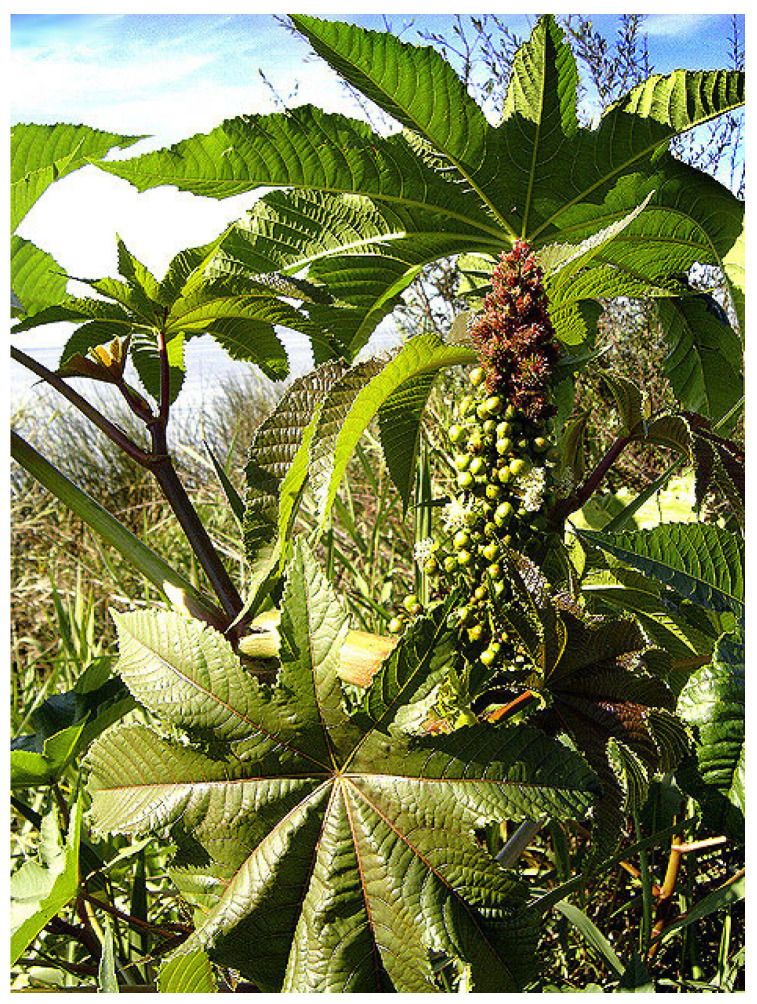
Castor oil plant with fruits containing seeds [16].

The matrix used in this work was castor oil polyurethane. Castor oil (*Ricinus communis*) is a plant of the Euphorbiaceae family, native to South Asia (see Figure 2). An oil is produced from the seeds that has a hydroxyl (OH) linked to the carbon chain. There is no other commercially produced vegetable oil with this property and polyurethane is obtained from this oil. The main countries producing castor oil are India (74%), China (13%), Brazil (6.1%), and Mozambique (2.5%) [16]. In Brazil, the only manufacturer of castor oil polyurethane is Imperveg in the state of São Paulo.

Sandwich panels are a type of structural composite in which the core is made of a material less dense than the faces. They are designed to be lightweight beams with high rigidity, consisting of two faces bonded to a core using an adhesive [17]. One of the most commonly used core structures is the honeycomb. Honeycombs belong to the class of cellular materials, characterized by ‘voids’ separated by solid walls, and are notable for their low relative density. The energy absorption capacity of a honeycomb is strongly influenced not only by the mechanical properties of the material composing the honeycomb and the thickness of its walls but also by the cell configuration [18]. One of the processes that can be used to manufacture honeycombs is additive manufacturing, specifically through Fused Deposition Modeling (FDM) 3D printing [19].

Additive manufacturing (AM), also known as 3D printing (a commonly used term), is a manufacturing process characterized by the addition of successive layers of material based on a 3D geometric model [20]. Additive manufacturing through FDM 3D printing allows for the creation of parts tailored to specific applications, with low tooling costs, design freedom, and faster prototyping cycles. In engineering, this method of manufacturing is widely used and is expected to experience strong growth in the coming years. The 3D printing market was valued at $12.6 billion in 2020, and forecasts for 2026 predict that the market will reach $37.2 billion [20].

This study focused on a sandwich composite structure using sisal fiber composite faces in a castor oil-based polyurethane matrix and a honeycomb structural core, printed in PETG (polyethylene terephthalate glycol) and PLA (polylactic acid) using a 3D printer. The work comprises two parts: the first part aims to determine the optimal chemical treatment for the sisal fiber to achieve the best mechanical properties, particularly tensile strength; the second part analyzes the sandwich composites, applying the most effective treatment identified.

## 2. Materials and Methods

Firstly, the study focused on determining the optimal chemical treatment to achieve the strongest adhesion between the sisal fibers and the matrix, so that this treatment could later be applied to the fibers in the plates or faces of the sandwich composites. This study of the influence of alkaline treatment on sisal fibers to increase the fibers’ adhesion to castor oil polyurethane was carried out because there are no relevant studies worldwide on the use of this castor oil polyurethane resin and the adhesion of vegetable fibers to this polyurethane. The study varied the concentrations of sodium hydroxide and the immersion time of the fibers in the solution. Subsequently, tensile test specimens were manufactured using a castor oil-based polyurethane matrix to identify which treatment resulted in the highest tensile strength, thereby determining the most effective treatment.

The materials used in this initial study included: sisal fibers supplied by FIBRAEX, located in Conceição do Coité, Bahia, Brazil; sodium hydroxide provided by the Chemistry Laboratory of the Escola Superior de Tecnologia (UEA/EST) in Manaus, Brasil; and castor oil polyurethane supplied by Imperveg^®^ (Brazil) in São Paulo. According to the supplier’s specifications, 0.2% to 0.5% of an anti-bubble additive (Siladit 53) supplied by REDELEASE should be used relative to the total weight of the mixture.

When we subject plant fibers to alkaline treatment, we expect to eliminate linin and ash and increase the surface roughness of the fiber to achieve greater adhesion of the fiber to the polymer and thus increase mechanical resistance. There is no specific treatment in the literature that always gives the best results, because it depends on many factors such as type of fiber, age of the plant, soil where the plant grew, type of resin, etc. Therefore, it is important to always carry out this study as this work used a matrix that has been little researched worldwide (castor oil polyurethane).

**Figure 3:**
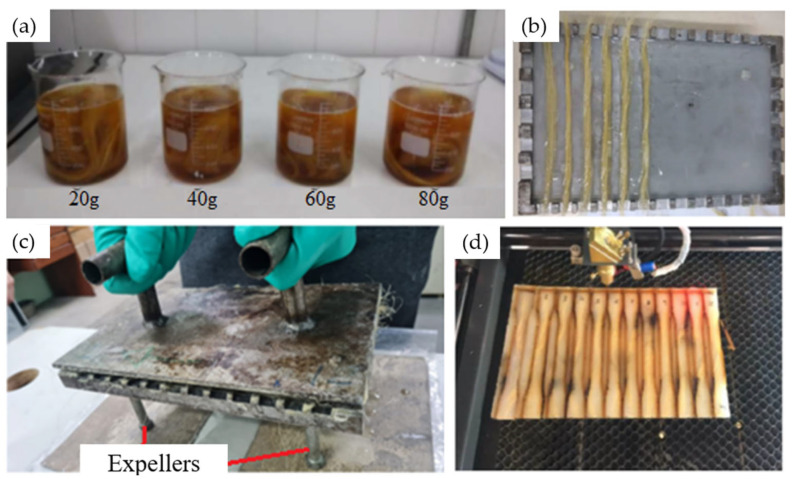
Manufacturing test specimens: (**a**) the chemical treatment of the fibers, (**b**) the placement of the fibers and resin inside the mold, (**c**) closing the mold, (**d**) the cutting of the test specimens with a laser.

Before undergoing chemical treatments with sodium hydroxide, the sisal fibers were processed to remove impurities, washed with running water, and air-dried at room temperature for 24 h. Four different concentrations of sodium hydroxide were used: 2.5%, 5%, 7.5%, and 10% (by weight), corresponding to 20 g, 40 g, 60 g, and 80 g of sodium hydroxide, respectively, in 800 mL of distilled water, as shown in Figure 3a. A fiber-to-solution ratio of 3:8 was maintained in all cases [11,21]. The fibers were immersed in the solution for 1, 4, 8, and 12 h [11,21]. After each immersion period, the fibers were thoroughly washed with running water, followed by distilled water to stabilize the pH, then air-dried at room temperature for 24 h and subsequently dried in an oven at 60 °C for 8 h. Finally, when preparing the composites in the mold, they were placed in an oven at 100 °C for one hour to completely remove moisture. The oven used was a QUIMIS model 0317M–72 (São Paulo, Brazil), located in the Materials Laboratory of EST/UEA.

To manufacture the test specimens, composite plates were made using a metal mold with internal dimensions of 225 mm × 155 mm × 3 mm. Before starting the manufacturing process, a release wax (Tec Glaze-N), supplied by REDELEASE (São Paulo, Brazil), was applied to the internal surface of the mold to facilitate the extraction of the composite. The test specimens were created by mixing the polyurethane and hardener in a 100:50 ratio, along with 0.5% of the anti-bubble additive in a beaker, according to the manufacturer’s recommendations. The mixture was stirred manually for 15 min. A portion of the resin was then poured into the mold, followed by the placement of the fibers as shown in Figure 3b, and finally, the remaining resin was added. Before closing the mold, isopropyl alcohol was sprayed on the surface to help eliminate any potential bubbles [12,22]. The mold was then closed, and cold pressure was applied to the top lid.

**Figure 4:**
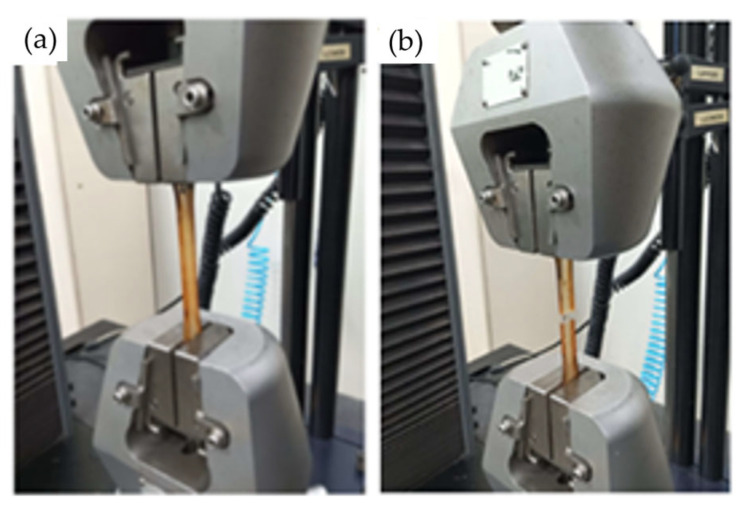
Tensile tests: (**a**) the start of the application of the load on the traction machine, (**b**) the end of the test at the moment of rupture.

The mold was kept under pressure for 24 h during the initial curing process. Afterward, the composite sheet was removed from the mold using the extraction screws (Figure 3c) and placed in an oven at 80 °C for 8 h to complete the curing process and enhance the strength of the composite. Once cured, the composite sheet was taken to a laser cutting machine (CNC Laser Router model VS6040) provided by the Machining Laboratory of the Mechanical Engineering Coordination of the UEA, Manaus, Brazil to cut the test specimens according to the dimensions specified by the tensile testing standard [23] (Figure 3d). All test specimens contained the same amount of fiber (20% by weight) to study the effect of the chemical treatment. The specimens, after being cut to the standard dimensions for tensile testing, were left at room temperature for 24 h to relieve internal stresses and were then taken to the EST/UEA Mechanical Testing Laboratory for tensile testing. The goal was to determine which specimens could withstand the highest tensile stress, thereby indicating the most effective treatment. All tensile tests were conducted using a model 5984 universal electromechanical testing machine with a 150 kN load cell, provided by EST/UEA, in the materials engineering course laboratory (see Figure 4a,b). These tests followed a standard speed of 5 mm/min. The combination of 4 different concentrations of sodium hydroxide in dissolution and 4 different immersion times of fiber in dissolution resulted in a total of 16 specimen types. Since 4 replicates were performed for each case to obtain average values, the total number of specimens was 64.

**Figure 5:**
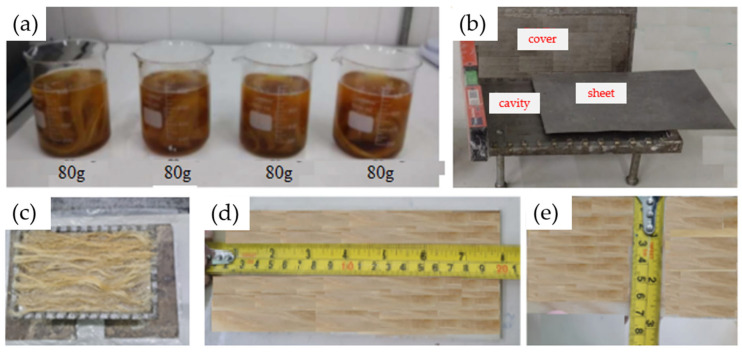
Manufacturing of the composite faces or sheet: (**a**) the alkaline treatment, (**b**) the mold for the manufacture of the sisal/epoxy composite sheets, (**c**) the placement of the fibers and resin inside the mold, (**d**) face length, (**e**) face width.

Therefore, since the treatment with 10% by weight of sodium hydroxide concentration in dissolution and 4 h of fiber immersion time was the highest average tensile stress, this treatment was used for the fibers in the manufacture of the sandwich composites (as you can see in Table 2 and Figure 5). The same materials described earlier (sisal fibers and a castor oil polyurethane matrix) were used to create the faces of the sandwich composites, while PETG (polyethylene terephthalate glycol) and PLA (polylactic acid) 3D printer filaments were used to manufacture the cores.

The initial processing, washing, and drying of the sisal fibers were carried out as previously described, with the exception of the chemical treatment. In this case, only the treatment determined by the earlier results was used: 10% by weight of sodium hydroxide and 4 h of fiber immersion in the solution, as shown in Figure 5a. The fibers were then washed and dried following the same procedure as before.

The manufacturing process of the sisal composite sheets in a castor oil polyurethane matrix followed a similar procedure to that previously described, with the exception that a 1 mm thick metal sheet was placed in the mold cavity to produce a 2 mm thick composite sheet, as the mold cavity is 3 mm deep (Figure 5b). The process of placing the fibers and resin was also similar, with the difference being that the fibers were uniformly distributed across the entire mold area rather than grouped by specimen, as carried out previously (Figure 5c). The curing process of the composite sheets was identical to the previously described method. After curing, the 2 mm thick composite sheets were cut into dimensions of 200 mm × 70 mm using a laser cutting machine, as shown in Figure 5d,e. A total of 8 faces were cut to fabricate 4 sandwich composites. The composite cores were then manufactured using a honeycomb structure with a 3D printer. Honeycomb cores are the most conventional geometry used in sandwich panel manufacturing. They are non-homogeneous cores with open cells in the transverse direction of the sheets, providing bidirectional support [24,25].

PETG polymer offers significant mechanical advantages over other polymers commonly used in additive manufacturing, such as ABS and PLA. Additionally, PETG can be obtained in a recyclable form, promoting the reuse of materials that would otherwise go to waste or even harm the environment. PLA (polylactic acid) is a linear, thermoplastic, semicrystalline or amorphous aliphatic polyester. It is a polymer synthesized from renewable sources like corn, potatoes, and sugarcane through bioconversion and polymerization. PLA has several noteworthy characteristics, including biocompatibility, biodegradability, and biological absorption, along with good mechanical and processability properties, thermal stability, and a low environmental impact [26,27].

**Figure 6:**
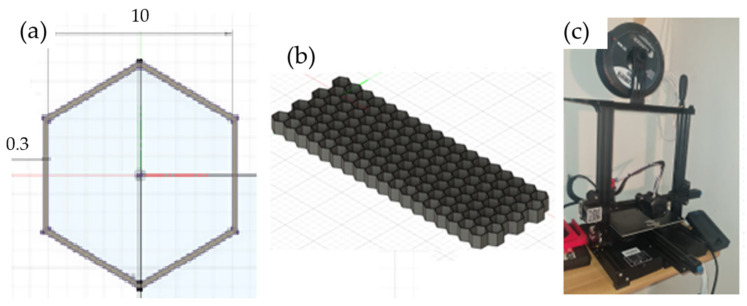
Printer configuration: (**a**) hexagon dimensions, (**b**) honeycomb model, (**c**) CREALITY ENDER 3 V2 printer model.

For the 3D modeling process, Fusion 360 software, a free licensed product by Autodesk, was used. To define the dimensions of the cores, three criteria were applied: the smallest possible layer thickness, two types of core heights, and a uniform hexagon size for all cores, as shown in Figure 6a,b. The wall thickness was set at 0.3 mm ± 0.05 mm, accounting for the horizontal expansion of the filament (Figure 6a). The core heights were defined as 10 mm and 15 mm, based on the commercially available honeycomb cores [18,25]. After modeling all the cores, they were organized according to Table 2. The printer used was a CREALITY ENDER 3 V2, with a printing area of 220 mm × 220 mm and a maximum height of 250 mm, located in the EST-UEA Machining Laboratory of the Mechanical Engineering Coordination of the UEA, Manaus, Brazil, as shown in Figure 6c.

Before starting the printing process, several calibrations were performed on the printer, including E-STEP (configuration of the extruder stepper motor), Flow (the amount of material extruded), and Leveling (ensuring the print bed is flat and level at all four corners). Additionally, the temperatures of the printing nozzle and the bed were calibrated.

**Figure 7:**
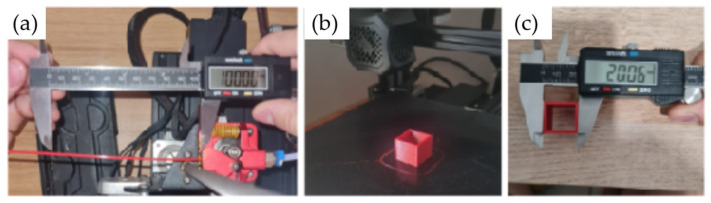
Printing parameters: (**a**) E–STEP calibration, (**b**) printing and (**c**) calibration cube measurement.

First, the extruder stepper motor was configured, which is responsible for moving the filament (E–STEP) in steps/mm, as can be seen in Figure 7a. To calibrate this, it was necessary to measure 100 mm of filament before the extruder and then ask the printer to deposit the 100 mm. If the stepper motor is calibrated, it will consume the 100 mm of filament; otherwise, a calculation based on the difference between the real and machine filaments must be performed. The calculated step was E = 98.73 steps/mm. The flow was then calibrated by printing a hollow cube with 20 mm sides to check whether the wall thickness was in accordance with the designed value. For this, the Ultimaker CURA slicing software version 5.8 was used. After printing, the cube was measured (See Figure 7b,c) and the values were compared with those configured by the software. The software’s default flow is 100% and the flow obtained was 100.9%, which is within the appropriate parameters.

The printer bed was leveled, and for this purpose it was necessary to direct the printing nozzle with height Z = 0, in the four corners of the bed, making the necessary height adjustments with the aid of the leveling nut. Finally, the nozzle and bed temperature were calibrated for the two materials. To calibrate the nozzle temperature, the temperature tower model was used, which consists of printing a tower with several types of temperature, until a temperature is achieved that the part obtains good quality. For the bed temperature values, the machine’s standard configuration were used. The temperatures determined for the nozzle were 205 °C for PLA and 240 °C for PETG, and for the bed they were 65 °C for PLA and 70 °C for PETG.

**Figure 8:**
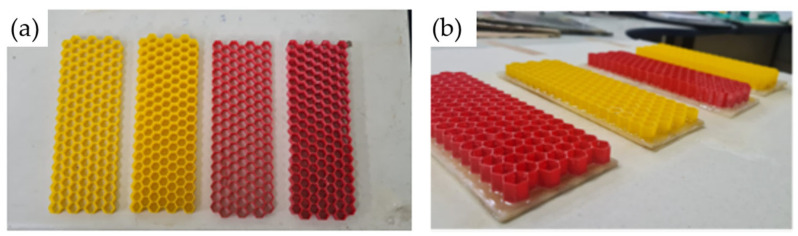
The manufacturing and gluing of the honeycombs. (**a**) The printing of the honeycombs and (**b**) the gluing of the first side.

All calibrations were first completed with PETG and then with PLA, as each material requires different settings. The slicing software, Ultimaker CURA^®^, which is available for free from Ultimaker, was also configured. All calibrated printing parameters were entered into the software. Four cores were printed for the test specimens, each following the dimensions of the 3D models (Figure 8). Table 3 provides the average dimensions, time, and weight for each printed core.

**Figure 9:**
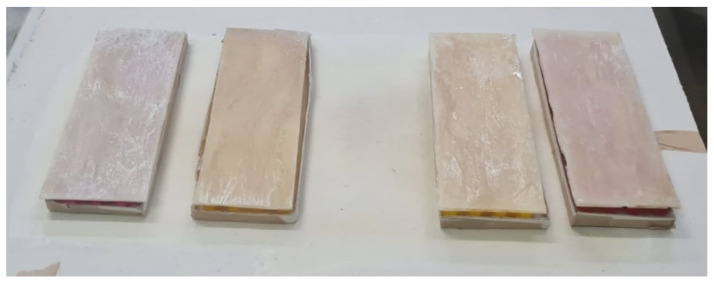
Top face bonding.

After printing, the honeycomb cores were glued to the faces of the sisal composite. The same castor oil polyurethane was used for gluing, but in smaller quantities: approximately 100 g of resin to 50 g of hardener and 0.75 g of anti-bubble additive. Approximately 55 g of this mixture was used for each face at each gluing stage. The process was divided into two steps: first, gluing the core to the lower face, and then gluing the upper face. After pouring the resin onto the faces, the core was placed centrally, and a weight was applied to ensure proper adhesion. The process was repeated after 48 h to glue the upper face (Figure 8 and Figure 9).

Subsequently, three-point bending tests were conducted on the sandwich composites. The tests were performed using the INSTRON 5984 machine at the P&D Materials Laboratory of EST/UEA (Manaus, Brasil). All specimens had dimensions of 200 mm × 70 mm, with 2 mm thick faces. The tests followed the ASTM standards [28,29] for three-point bending, with a test speed of 2 mm/min. The distance between the lower supports was 100 mm.

## 3. Results

**Figure 10:**
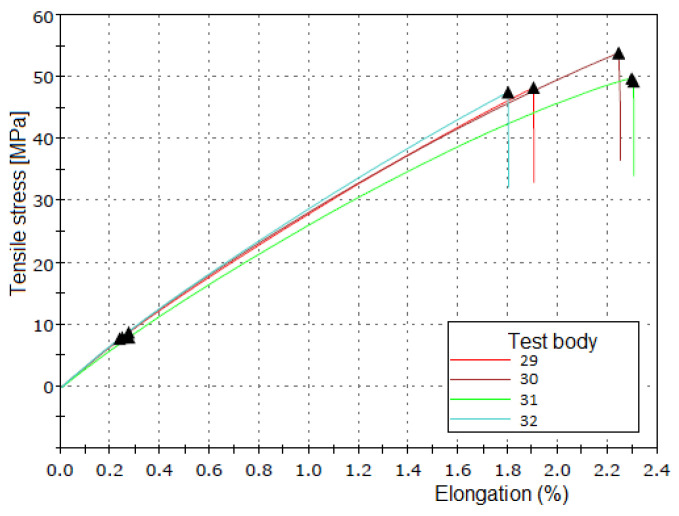
The graphs generated by the tensile testing machine for the test specimens with the highest tensile stress corresponding to an alkaline treatment with a 10% concentration and 4 h of immersion of the fiber in the dissolution.

Sixty-four tensile tests were performed and presented in groups of four tests, each corresponding to the replicates of a case study, resulting in 16 graphs, each displaying 4 stress–strain curves as shown in Figure 10. Table 4 presents the average tensile test results for each chemical treatment condition. The best results were obtained with a 10% by weight sodium hydroxide concentration and 4 h of immersion, consistent with findings reported in the literature [21,22,30]. Figure 10 shows the graph with the stress–strain curves corresponding to this chemical treatment, which resulted in the highest tensile stress (best treatment).

**Figure 11:**
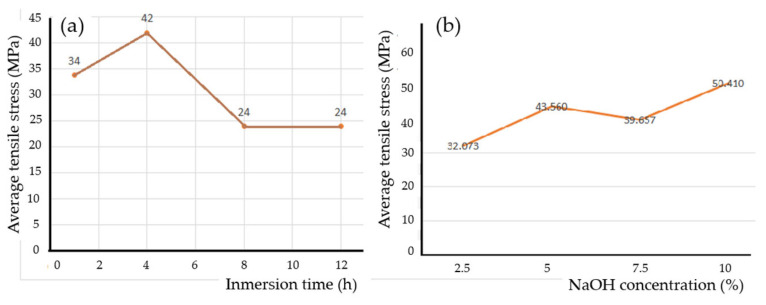
The influence of alkaline treatment on tensile stress: (**a**) the behavior of the average tensile stress as a function of immersion time, (**b**) the influence of NaOH concentration on tensile stress for an immersion time of 4 h.

There is no specific treatment in the literature that always gives the best results regarding alkaline treatment with NaOH because it depends on many factors such as the type of fiber, the age of the plant, the soil where the plant grew, the type of resin, etc. Therefore, it is important to always carry out this study and, in this work, this was performed was using a castor oil polyurethane matrix that has been little researched worldwide. As can be seen in Table 4 and Figure 11a, the most important immersion time (best tensile stress) result was 4 h. For this length of immersion time, whenever the sodium hydroxide content was increased, the tensile stress improved, as can be seen in Figure 11b. This behavior is due to the immersion time of 4 h being appropriate to produce an adequate influence to improve tensile stresses and immersion times of more than 4 h, such as 8 and 12 h, in addition to converting type I cellulose into type II, which can produce fiber deterioration and lead to a decrease in mechanical properties. It can be seen in the literature that the immersion time was the predominant factor in improving fiber adhesion to the matrix and the 4 h time was suggested in several previous studies [21,22,30].

The tensile stresses obtained in the traction tests on composites with sisal fibers with chemical treatment in castor oil polyurethane as a matrix decrease by 50% when compared with sisal composites with the same chemical treatments in an epoxy resin matrix, which indicates that composites in the castor oil polyurethane matrix are less resistant than the other matrix, but this is an important ecological matrix in terms of bioeconomy and sustainability [21,22].

**Figure 12:**
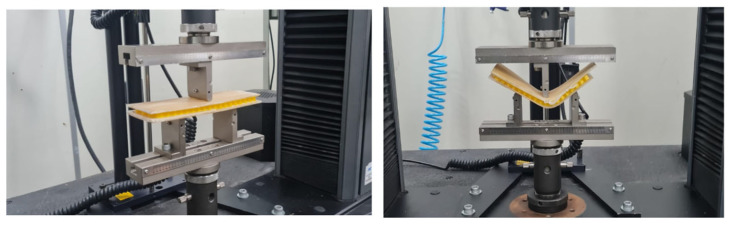
Three-point bending test of specimen 1.

**Figure 13:**
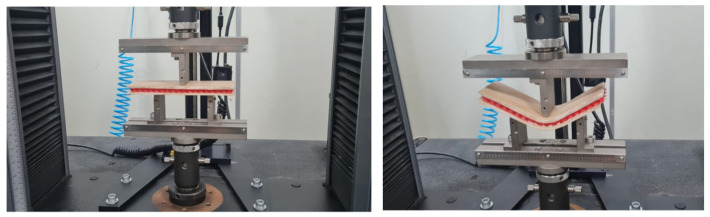
Three-point bending test of specimen 2.

**Figure 14:**
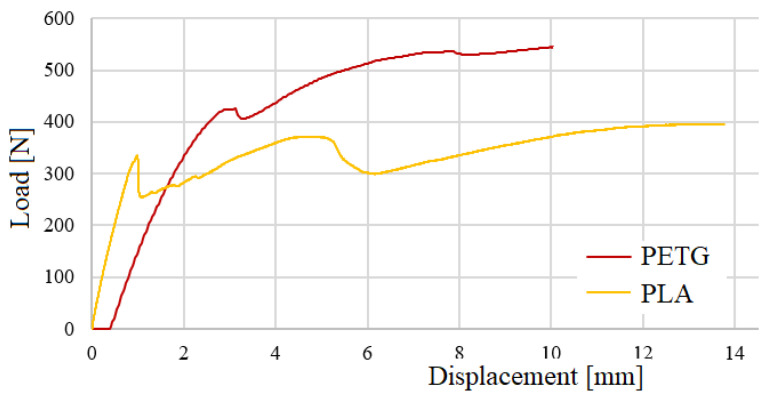
Bending load–displacement curves from the three-point bending tests for the 10 mm core height test specimens.

**Figure 15:**
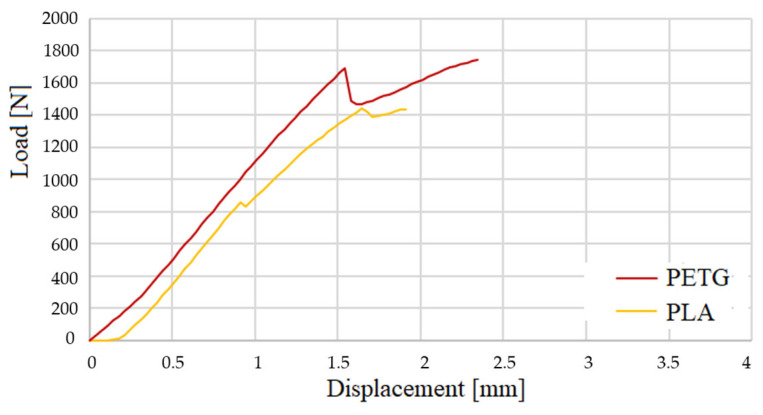
Bending load–displacement curves from the three-point bending tests for the 15 mm core height test specimens.

In Figure 12, three-point bending tests of test body 1 (PLA) can be observed, and in Figure 13, three-point bending tests of test body 2 (PETG) can be observed, corresponding to a Honney nucleus height of 10 mm. Figure 14 shows the bending force–displacement curves from the three-point bending tests of test bodies 1 and 2 (10 mm high PLA and PETG) generated by the machine, and Figure 15 shows the bending force–displacement curves from the three-point bending tests of the test bodies 3 and 4 (15 mm high PLA and PETG) generated by the machine.

The results of the three-point bending tests of the sandwich composites generated by the INSTRON testing machine are shown in Table 5 and Figure 14 and Figure 15. When analyzing the results in relation to core height, it was observed that composites with a 15 mm core supported 3.3 to 3.6 times more load than those with a 10 mm core. This can be explained by several reasons related to structural mechanics and the distribution of tensions in composite materials. For example, the resistance to flexion of a sandwich panel is influenced by the moment of inertia, which depends on the distance between the outer faces of the panel. By increasing the height of the core from 10 mm to 15 mm, this distance is increased, which increases the moment of inertia of the panel. A greater moment of inertia improves the rigidity of the panel, allowing it to withstand greater loads without deforming excessively.

On the other hand, in a sandwich panel, the outer faces support the greater part of the compression and traction tensions when the panel undergoes flexion, and by increasing the height of the core the faces are more separated. This means that the panel can support a greater load before failure occurs.

**Figure 16:**
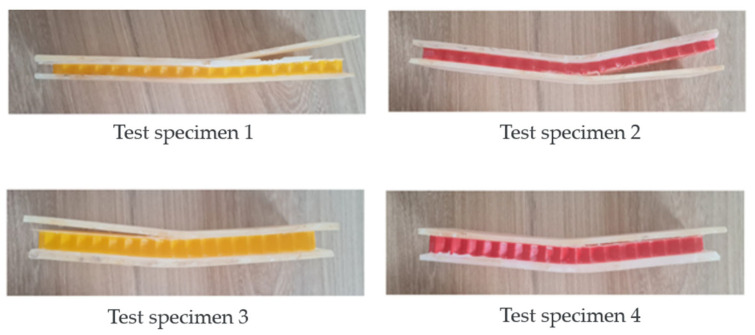
Specimens (1–4) After Bending Tests.

As illustrated in Figure 16, there was no rupture of the faces, but detachment of the core occurred, because the core is not adequately supported by the faces, because the core material does not have sufficient shear resistance under low loads due to the lack of continuity between the core and the faces, or the presence of areas of lower adherence, where the core can shift when a load is applied. If there is a significant difference in stiffness between the sides of the panel and the core, the sides could deform differently to the core under load, which would lead to relative displacement of the core. This is especially relevant if the nucleus is less rigid or more flexible than the faces. The data from the tests enabled the analysis and comparison of the stress and deformation of the specimens. The results align with findings from other previously published works [31,32].

**Figure 17:**
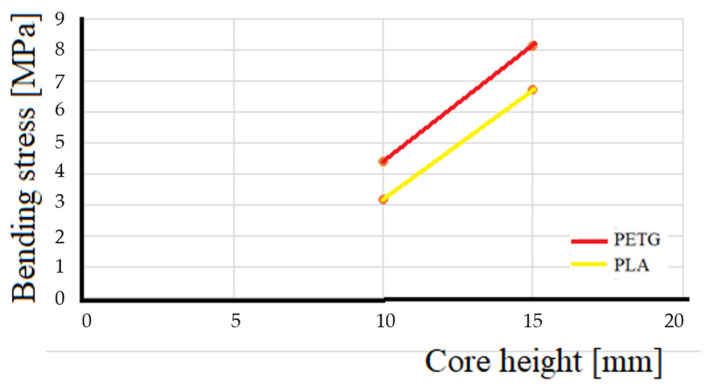
Influence of material and honeycomb height on maximum bending stress.

In all tests, PETG was stronger, generally 21% to 32% stronger, than PLA (Figure 17). The PETG combines high resistance with good ductility, which allows it to withstand greater loads before failing in comparison to PLA which, even though it has good mechanical properties, is more rigid and fragile (see graph in Figure 14) so is more likely to deform under load before reaching the same resistance as the PETG. Another reason is that PLA is lighter due to its lower density and this lightness translates into lower stiffness and overall resistance of the material. In sandwich-type structures, a higher density, like that of PETG, contributes to a better distribution of loads and a greater resistance to compression and flexion, which explains why PETG is stronger. Also, during 3D printing, PETG tends to have better adhesion between layers than PLA, which reduces the possibility of gaps between layers and improves the general resistance of the core. This could also contribute to the greater strength and lower deformation observed in PETG cores.

The sandwich composites evaluated in this work have the same geometry, which was determined using the standards and corresponding bibliography [31,32]. Only the core height was varied by 5 mm between one model and the next for each material in order to verify the influence of the variation in core height on the bending stress. The slope of the PLA curve, as can be seen in Figure 17, is 40.7 degrees, and the slope of the curve for PETG is 43 degrees. This means that increases in the core height of the sandwich composites produce increases in the bending stress in both materials, mainly due to the increase in the moment of inertia, but in the case of PETG, this increase is greater than that of PLA. The results of the work coincide with the simple theoretical analysis based on the bending formula of a simply supported beam by HIBBELER, R.C. 2010 [33] where: Vmax is the maximum deflection, P is the maximum load, L is the distance between the lower supports of the three-point bending test, E is the deformation modulus, I_y_ is the moment of inertia, b is the width of the panel, and h is the height of the sandwich panel. Thus, the material with greater resistance (greater E) will have less deformation and the cores with greater height will also have less deformation, as can be seen in Table 5.

$$v_{máx}=-\frac{PL^{3}}{48EI}\;\;\;\;\;I_{y}=\frac{bh^{3}}{12}$$

## 4. Conclusions

The main objective of this research was to evaluate sandwich composites of ecological materials using sisal fiber faces in a castor oil polyurethane matrix and a PLA and PETG honeycomb core manufactured in a 3D printer. There are no published works in the literature on the adhesion of vegetable fibers to castor oil polyurethane, nor on the use of castor oil polyurethane on the faces of sandwich composites. Here, a study was carried out on sandwich composites with sisal fiber faces in a castor oil polyurethane matrix with good results. The results of the tensile tests show that the alkaline treatment with sodium hydroxide improves the adhesion of sisal fibers to the castor oil polyurethane, with the best treatment being 10% sodium hydroxide in the dissolution and 4 h of fiber immersion time in the dissolution. It was also found that composites of vegetable fibers in a castor oil polyurethane matrix had lower tensile stress values (50%) than composites in an epoxy resin matrix. However, castor oil polyurethane is an important ecological matrix in terms of bioeconomy and sustainability.

The results of the three-point bending tests showed that the sandwich composites with a PETG core had better bending stress results than the composites with a PLA core and showed that the sandwich composites with a core height of 15 mm had better bending stress results than the composites with a core height of 10 mm for two materials. Increasing the core height of the sandwich composite produces a greater increase in bending stress for PETG than for PLA.

The results were consistent with those of previous studies in the literature. The honeycomb structure proved to be a very strong arrangement with a low density, occupying only 5.4% of the available volume between the faces, which makes it suitable for applications that may require the passage of internal materials such as pipes and cables. Additionally, these cores can be used in applications that require low density, such as in the aeronautical and naval industries.

As a result, it was found that castor oil polyurethane can be used in composites with vegetable fibers, obtaining a completely ecological composite from renewable sources and using cores manufactured in 3D printers with PLA filaments that are also obtained from renewable sources and PETG obtained from recycling.

## Figures and Tables

**Table 1:** Properties and chemical compositions [4,14].

Physical properties						
Density (g/cm^3^)		Internal porosity %		Diameter (μm)		Fiber length (mm)
1.45		17		150 to 200		80 to 120
Chemical composition						
Cellulose (%)		Hemicellulose (%)		Lignin (%)		Waxes (%)
50–78		10–20		8–12		2
Mechanical properties						
Tensile stress MPa			Elasticity Modulus (GPa)		Elongation (%)	
480			40		5 to 14	

**Table 2:** Printed Core Data.

Type	Material	Wall Thickness	Core Height	Hexagon Inner Width
1	PLA	0.3 mm ± 0.05	10 mm ± 0.05	10 mm ± 0.05
2	PETG	0.3 mm ± 0.05	10 mm ± 0.05	10 mm ± 0.05
3	PLA	0.3 mm ± 0.05	15 mm ± 0.05	10 mm ± 0.05
4	PETG	0.3 mm ± 0.05	15 mm ± 0.05	10 mm ± 0.05

**Table 3:** Parameters of each printed honeycomb.

Type	Material	Wall Thickness (mm)	Core Height (mm)	Hexagon Inner Width (mm)	Weight (g)
1	PLA	0.39	9.88	10.02	10.4
2	PETG	0.38	9.87	10.03	11.3
3	PLA	0.38	14.93	10.02	16.2
4	PETG	0.39	14.87	10.03	17.3

**Table 4:** Test specimens, chemical treatment data, and average results of the values generated by the tensile testing machine.

Test Specimen	NaOH Concentration (%)	Immersion Time (h)	Elasticity Modulus (MPa)	Elongation (%)	Tensile Stress (MPa)
CP 1–4	2.5	1	3052.080 ± 1	1.877 ± 1.7	44.867 ± 0.8
CP 5–8	5.0	1	3031.667 ± 1.2	1.570 ± 1.1	36.333 ± 1.3
CP 9–12	7.5	1	2870.753 ± 0.8	1.557 ± 1.9	37.103 ± 1
CP 13–16	10.0	1	2872.157 ± 1.5	0.787 ± 0.8	17.140 ± 1.8
CP 17–20	2.5	4	2853.547 ± 1.4	1.330 ± 1.3	32.073 ± 2.2
CP 21–24	5.0	4	3088.103 ± 1.1	1.830 ± 2.1	43.560 ± 1.9
CP 25–28	7.5	4	3299.037 ± 1.2	1.623 ± 1.4	39.657 ± 1.1
CP 29–32	10.0	4	3168.073 ± 0.8	2.110 ± 2.2	50.410 ± 1
CP 33–36	2.5	8	3222.050 ± 1.2	1.420 ± 1.6	34.547 ± 2.3
CP 37–40	5.0	8	2997.180 ± 0.9	1.223 ± 1.2	30.110 ± 1.7
CP 41–44	7.5	8	3061.000 ± 1	0.807 ± 1.4	14.510 ± 1.1
CP 45–48	10.0	8	3171.513 ± 1.3	0.833 ± 0.8	19.340 ± 2.4
CP 49–52	2.5	12	3220.137 ± 1.1	0.963 ± 2	21.450 ± 2.1
CP 53–56	5.0	12	3379.093 ± 1.8	0.923 ± 1.8	24.777 ± 1.5
CP 57–60	7.5	12	3323.750 ± 0.8	1.267 ± 1.7	28.080 ± 2
CP 61–62	10.0	12	3258.440± 1.4	0.980 ± 0.9	23.603 ± 1.2

**Table 5:** Results of three-point bending tests generated by the INSTRON testing machine for all specimens.

Type	Core Material	Core Height (mm)	Maximum Load [N]	Bending Stress at Maximum Load [MPa]	Bending Strain [%]	Modulus of Elasticity [GPa]
1	PLA	10	396.13	3.18	13.32	0.023
2	PETG	10	525.1	4.38	9.68	0.043
3	PLA	15	1443.91	6.73	2.41	0.280
4	PETG	15	1744.62	8.13	3.00	0.342

## Data Availability

Data are contained within the article.
